# Evaluation of the Limit of Detection of Bacteria by Tandem Mass Spectrometry Proteotyping and Phylopeptidomics

**DOI:** 10.3390/microorganisms11051170

**Published:** 2023-04-29

**Authors:** Charlotte Mappa, Béatrice Alpha-Bazin, Olivier Pible, Jean Armengaud

**Affiliations:** 1 Université Paris-Saclay, CEA, INRAE, Département Médicaments et Technologies pour la Santé (DMTS), SPI, 30200 Bagnols-sur-Cèze, France; 2 Laboratoire Innovations Technologiques pour la Détection et le Diagnostic (Li2D), Université de Montpellier, 30207 Bagnols-sur-Cèze, France

**Keywords:** bacteria, detection, mass spectrometry, shotgun proteomics, proteotyping

## Abstract

Shotgun proteomics has proven to be an attractive alternative for identifying a pathogen and characterizing the antimicrobial resistance genes it produces. Because of its performance, proteotyping of microorganisms by tandem mass spectrometry is expected to become an essential tool in modern healthcare. Proteotyping microorganisms that have been isolated from the environment by culturomics is also a cornerstone for the development of new biotechnological applications. Phylopeptidomics is a new strategy that estimates the phylogenetic distances between the organisms present in the sample and calculates the ratio of their shared peptides, thus improving the quantification of their contributions to the biomass. Here, we established the limit of detection of tandem mass spectrometry proteotyping based on MS/MS data recorded for several bacteria. The limit of detection for *Salmonella bongori* with our experimental set-up is 4 × 10^4^ colony-forming units from a sample volume of 1 mL. This limit of detection is directly related to the amount of protein per cell and therefore depends on the shape and size of the microorganism. We have demonstrated that identification of bacteria by phylopeptidomics is independent of their growth stage and that the limit of detection of the method is not degraded in presence of additional bacteria in the same proportion.

## 1. Introduction

Rapid detection and identification of microorganisms is of utmost importance for the clinical microbiology laboratory as well as for the food industry, water suppliers, environmental applications, and microbial biotechnology. It is also an important goal to rapidly respond to bioterrorism threats and prevent the risk of dissemination of novel biological agents. Over the past decade, mass spectrometry has become a widespread technique for pathogen identification [1,2]. Indeed, whole-cell MALDI-TOF mass spectrometry, based on a comparison between experimental *m*/*z* profiles of basic, low-molecular-weight proteins of bacteria and a comprehensive database of spectra acquired under the same conditions, has revolutionized the clinical microbiology laboratory [3]. This approach can be refined with defining specific biomarkers [4,5]. For example, *Francisella tularensis* subspecies can be identified using specific biomarkers detected by MALDI-TOF mass spectrometry and previously defined by proteogenomics [6]. Furthermore, this approach can even be used to identify environmental bacteria for which the database is generally still poorly documented and to screen new isolates [7,8].

However, in the whole-cell MALDI-TOF mass spectrometry methodology, a single colony must first be obtained on an agar plate, which implies an overnight culture in most cases. In addition, the approach is not applicable to mixtures of microorganisms and it will be difficult to identify new environmental species that have not been previously recorded in the database. Proteotyping bacteria by tandem mass spectrometry analysis of trypsin-generated peptides from extracted proteins has proven to be a possible alternative for classification and identification of microorganisms [9]. To this end, several methodologies for interpreting MS/MS spectra to identify microorganisms have been proposed [10]. The pioneers in this field have tested a database restricted to the annotated genomes of 87 bacteria to interpret MS/MS datasets obtained with model bacteria. In this case, identification is based on unique peptides that are specific for a single organism [11]. A more complex database of 170 fully sequenced bacterial genomes was also proven successful [12], but this approach is not suitable for giant databases meant to represent the real microbial diversity, which will inevitably decrease the number of theoretical specific peptides per microorganism [13]. Alternatives have been proposed, such as the TCUP method that relies on specific peptides tailored to characterize taxonomic composition and capable of typing multiple clinically relevant species [14]. Peptide matching against a reference database derived from metagenomics sequencing is also used for bacterial identification and quantification of biomass contributions in metaproteomics [15]. In this methodology, the analysis is either restricted to a set of representative species that do not have significant overlap or is performed by allocating peptide results to the lowest common ancestor [16]. Peptide-intensity-weighted proteome abundance similarity correction based on the calculation of a matrix containing the pairwise similarities of the reference proteomes from the identified bacteria has been proposed to improve the quantification of biomass contributions in metaproteomics [17]. Recently, a new strategy has been shown to give very reliable quantification of biomass contributions after estimating the phylogenetic distances between the organisms present in the sample and calculating their ratio of shared peptides [18]. Phylopeptidomics has been able to distinguish with high accuracy the ratio of two closely related pathogens of clinical interest, *Salmonella bongori* and *Shigella flexneri*. This approach also performs well in identifying taxa present in the sample by combining unique taxon-specific peptide sequences with shared peptides that can be inferred by deconvolution of the interpreted signals from tandem mass spectrometry [18].

Rapid detection and identification of microorganisms by tandem mass spectrometry is gaining momentum [19,20], as the methodology has been shown to be high-throughput [21] and is important for clinical diagnostics. The potential of the methodology was recently illustrated by studies on ancient samples [22,23]. For such type of samples, the method was renamed paleoproteotyping. Many alternatives have been proposed to interpret the data [24,25,26,27,28]. Unfortunately, little data are available to date on the detection limit of clinically relevant bacteria by tandem mass spectrometry. The detection limit by MALDI-TOF has been documented for culture-independent detection of pathogens from urine samples [29]. In this case, 1 × 10^5^ to 1 × 10^6^ colony-forming units per mL were required. For the identification of yeast from positive blood cultures, the limit of detection was established at 5.9 × 10^5^ cfu per mL [30]. The use of specific surfactants improved the detection limit of MALDI-TOF to 1 × 10^3^ to 1 × 10^4^ cfu per mL of bacterial suspension [31]. Targeted proteomics coupled with immunopurification of bacteria has proven to be an effective method when targeting a specific bacterium [32]. The detection limit for identification of *Yersinia pestis* based on specific peptides monitored by selected reaction monitoring was 2 × 10^4^ cfu per mL in tap water or milk. To the best of our knowledge, such a detection limit for various microorganisms is not yet available for proteotyping by tandem mass spectrometry based on the shotgun proteomics principle.

Here, we established the detection limit of bacteria using phylopeptidomics to interpret MS/MS data recorded by a high-resolution LTQ-Orbitrap XL instrument (Thermo) over a 60 min gradient. We also demonstrated that the identification of bacteria by phylopeptidomics is independent of their growth stage and that the detection limit of the method is not degraded in the presence of additional bacteria.

## 2. Materials and Methods

### 2.1. Microorganisms and Microbial Cultures

The strains *Salmonella bongori* NCTC 12419, *Deinococcus proteolyticus* MRP DSM 20540, and *Bacillus thuringiensis* ATCC 10792 were obtained from the Pasteur collection, the DSM collection, and the ATCC collection, respectively. *S. bongori* was grown overnight in liquid TSB medium under aerobic conditions with vigorous shaking (140 rpm) at 30 °C in a BSL2 safety laboratory. *D. proteolyticus* and *B. thuringiensis* were grown in the same conditions except that liquid LB medium was used. Cell densities were evaluated by means of optical density (OD) measured at 600 nm. Liquid cultures of 100 mL were inoculated with overnight cultures to obtain an initial OD at 600nm of 0.008. These liquid cultures were incubated at 30 °C until OD_600nm_ reached 0.1. Then, a serial dilution from 10 to 10 was performed and volumes of 100 µL of each of these dilutions were spread on LB medium agar plate. The plates were incubated overnight at 30 °C and the colonies were then counted to establish the relationship between number of colony forming units and OD_600nm_. Several samples of 1 mL of cell suspension at OD_600nm_ = 0.1 (exponential phase) were harvested and immediately centrifuged at 16,000× *g* for 5 min at room temperature. The resulting supernatants were removed, and the cell pellets were subjected to another round of centrifugation for 2 min to remove any residual liquid. Each wet bacterial pellet was dissolved in 1 mL of sterile phosphate-buffered saline (PBS) buffered at pH 7.4. Each of these cell suspensions was further diluted in order to obtain tubes of 1.0 mL of suspension at 4 × 10^3^ cfu/mL, 8 × 10^3^ cfu/mL, 1 × 10^4^ cfu/mL, 2 × 10^4^ cfu/mL, 4 × 10^4^ cfu/mL, and 8 × 10^4^ cfu/mL. A series of 1:1 mixtures of *S. bongori* and *D. proteolyticus* was prepared by mixing the corresponding number of bacteria prepared in PBS to get a final concentration of 2 × 10^4^ cfu/mL, and 2 × 10^5^ cfu/mL. For *S. bongori*, cells harvested at the stationary phase (overnight culture) were also prepared in similar conditions.

### 2.2. Sample Preparation for Shotgun Proteomics

The bacterial samples were precipitated with tricholoroacetic acid by adding to the 1 mL cell suspension a volume of 250 µL of trichloroacetic acid (Sigma Aldrich, Saint-Quentin-Fallavier, France) prepared at 50% (w/vol) and centrifuged at 16,000× *g* for 5 min at room temperature. The supernatants were removed and the pellets were again centrifuged for 2 min to remove any residual liquid. Each pellet was dissolved in 60 µL of LDS1X sample buffer (Invitrogen, Villebon sur Yvette, France) consisting of 106 mM Tris/HCl, 141 mM Tris base, 2% lithium dodecyl sulfate, 10% glycerol, 0.51 mM EDTA, 0.22 mM SERVA Blue G250, 0.175 mM phenol red, buffered at pH 8.5, and supplemented with 2.5% beta-mercaptoethanol. Samples were heated at 99 °C for 5 min (Eppendorf, Montesson, France) and then subjected to sonication in an ultrasonic bath (VWR ultrasonic cleaner, VWR, Rosny-sous-Bois, France) for 5 min to dissolve all the biological aggregates. The samples were transferred to tubes containing 50 mg of silica beads and subjected to bead-beating with a Precellys instrument (Bertin technology, Montigny-le-Bretonneux, France) operated at room temperature at 7800 rpm for 10 cycles of 20 s separated by 30 s pauses. After cell disruption, the tubes were centrifuged at 16,000× *g* for 40 s. The resulting supernatants were transferred into new tubes and heated at 99 °C for 5 min. A volume of 25 µL of protein extract was loaded onto a NuPAGE 4–12% Bis-Tris gel (Invitrogen). The proteins were subjected to a short denaturing electrophoresis migration of 5 min at 200 V in MES/SDS 1X running buffer (Invitrogen) and then stained with Simply Blue Safestain (Invitrogen). Each polyacrylamide band containing the whole soluble proteome of the sample was sliced from the gel, reduced with dithiothreitol and treated with iodoacetamide to modify cysteines as described [33]. The gel pieces were then subjected to in-gel trypsin proteolysis with trypsin in the presence of 0.01% ProteaseMAX detergent (Promega, Charbonnières-les-Bains, France) as described [34].

### 2.3. Liquid Chromatography and Tandem Mass Spectrometry

Peptides were analyzed with an LTQ-Orbitrap XL hybrid mass spectrometer (Thermofisher, Villebon sur Yvette, France) coupled to an ultimate 3000 nanoLC system (Thermo) operated in data-dependent mode essentially as previously described [35]. The digests (50 µL) were loaded with a specific large injection loop and desalted online on a reverse phase PepMap100 C18 µ-precolumn (5 µm, 100 Å, 300 µm i.d. × 5 mm, Thermofisher, Villebon sur Yvette, France) and resolved on a nano scale PepMap 100 C18 nano LC column (3 µm, 100 Å, 75 µm i.d. × 50 cm, Thermofisher) at a flow rate of 0.3 µL.min^−1^ with a gradient of CH_3_CN, 0.1% formic acid prior to injection into the ion trap mass spectrometer. Peptides were resolved using a 60-min gradient from 2.5% to 50% solvent B (0.1% HCOOH/20% H_2_O/80% CH_3_CN) against solvent A (0.1% HCOOH/100% H_2_O). A top 5 strategy was used for the acquisition of MS/MS essentially consisting in a full MS scan, followed by fragmentation and MS/MS scan on the 5 most abundant ion precursors. Full scan mass spectra were measured from *m*/*z* 300 to 1800 in the Orbitrap analyzer at 30,000 resolution. The MS/MS scans were triggered in the linear ion trap at a resolution of 10,000 with a minimum signal required set at 10,000, potential charge states of 2+ and 3+, and with a 10 s dynamic exclusion of previously selected ions.

### 2.4. MS/MS Data Analysis

MS/MS spectra were assigned in proteomics mode with dedicated protein sequence databases corresponding to the annotated genomes: *S. bongori* NCTC 12419, *D. proteolyticus* MRP DSM 20540, and *B. thuringiensis* ATCC 10792. The MS/MS spectra from the mixture of microorganisms were queried against the *S. bongori* NCTC 12419 and *D. proteolyticus* MRP DSM 20540 merged database. MS/MS spectra were assigned with the Mascot Daemon software version 2.6.1 (Matrix Science) with full-trypsin specificity, up to 1 missed cleavage allowed, static modifications of carbamidomethylated cysteine (+57.0215), variable oxydation of methionine (+15.9949), mass tolerance of 5 ppm on the parent ions, and MS/MS mass tolerance of 0.5 Da. All peptide matches with a peptide score below a query threshold set at *p* ≤ 0.05 and rank 1 were parsed. A protein was considered valid when at least two different peptides were detected. The false-positive rate for protein identification was estimated by a search with a reverse decoy database to be below 0.1% using the same parameters. The number of MS/MS spectra assigned per protein (spectral counts) was extracted for each sample. The normalized spectral abundance factor (NSAF) for each protein was calculated as the total spectral count divided by its molecular mass in kDa, as described previously [36]. Tfold change for comparative proteomics between growth phases was calculated after data normalization as recommended [37]. For assigning the MS/MS spectra in phylopeptidomics mode, the NCBInr database was used as previously described [18].

## 3. Results and Discussion

### 3.1. Phylopeptidomic Identification of S. bongori Is Independent of Growth Stage

**Figure 1:**
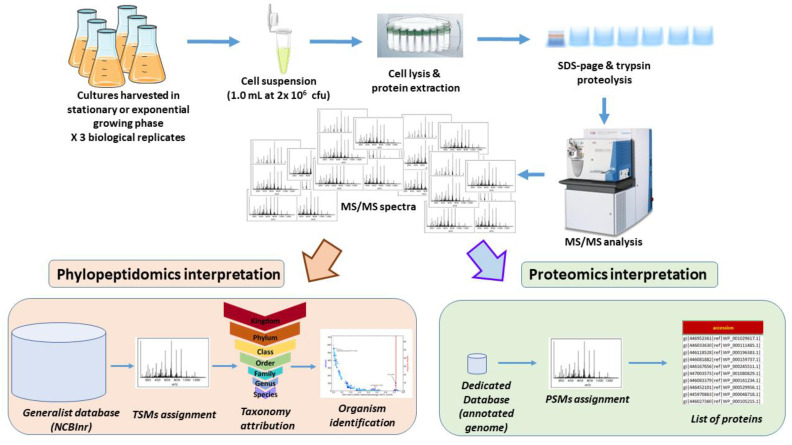
Experimental and data analysis strategy.

Figure 1 shows the strategy used for the analysis of *S. Bongori* harvested either in exponential phase or in stationary phase. Three independent biological replicates for both conditions with 1 × 10^6^ cfu were harvested, lysed, and the extracted proteins were subjected to trypsin proteolysis. The resulting peptides were characterized by tandem mass spectrometry after their separation by reverse phase liquid chromatography. As shown in Figure 1, two types of bioinformatics analysis were performed on the six raw files. First, a proteotyping assignment of the MS/MS spectra against a generalist database (NCBInr) was carried out to assign taxon-spectrum matches (TSMs) to identify the taxon present in the sample and list the specific peptide sequences of that taxonomical branch. Then, a more classical proteomic interpretation of the MS/MS spectra was performed using only the protein sequences of the annotated genome of the identified taxon.

Table 1 reports the number of MS/MS spectra recorded for each of the six samples containing a relatively small amount of biological material (1 × 10^6^ cfu, corresponding to the equivalent of 0.4 µL of a culture of bacteria at OD of 1.0 at 600 nm). On average, 3992 MS/MS spectra were recorded, yielding 282 TSMs when interpreted against NCBInr, filtered for taxonomical links between peptide sequences and taxa, and accounting for validated taxonomical units at the various possible taxonomical ranks. This low level of TSMs is due to the small amount of sample on the one hand, and the atypically inflated database used for the MS/MS assignation on the other. For the first replicate of *S. bongori* cells harvested in exponential phase, 197 unique taxon-specific peptide sequences were exclusively from the Bacteria superkindgdom. Of these, the vast majority belong to a single phylum: Proteobacteria, with 368 TSMs and 83 unique taxon-specific peptide sequences. Clearly, the number of unique taxon-specific peptide sequences decreases sharply (83 instead of 197) when moving from the taxonomical rank superkingdom to phylum, whereas this decrease is relatively mild (368 instead of 372) for TSMs. Peptide sequences of proteins that are highly conserved among all bacteria are generally not considered useful in terms of taxonomy even though they contribute to the bacterial signal. Since no other daughter taxon are represented in the dataset, the vast majority of TSMs are then assigned to Proteobacteria. The same reasoning was applied to lower taxonomical ranks. Taxon-specific peptides indicate the presence of a single class, Gammaproteobacteria; an order, Enterobacterales; a family, Enterobacteriaceae; a genus, *Salmonella*; and a species, *S. bongori*. Finally, the species *S. bongori* is validated by the presence of four unique taxon-specific peptide sequences, and a large number of TSMs (357), which is expected for such a species.

**Figure 2:**
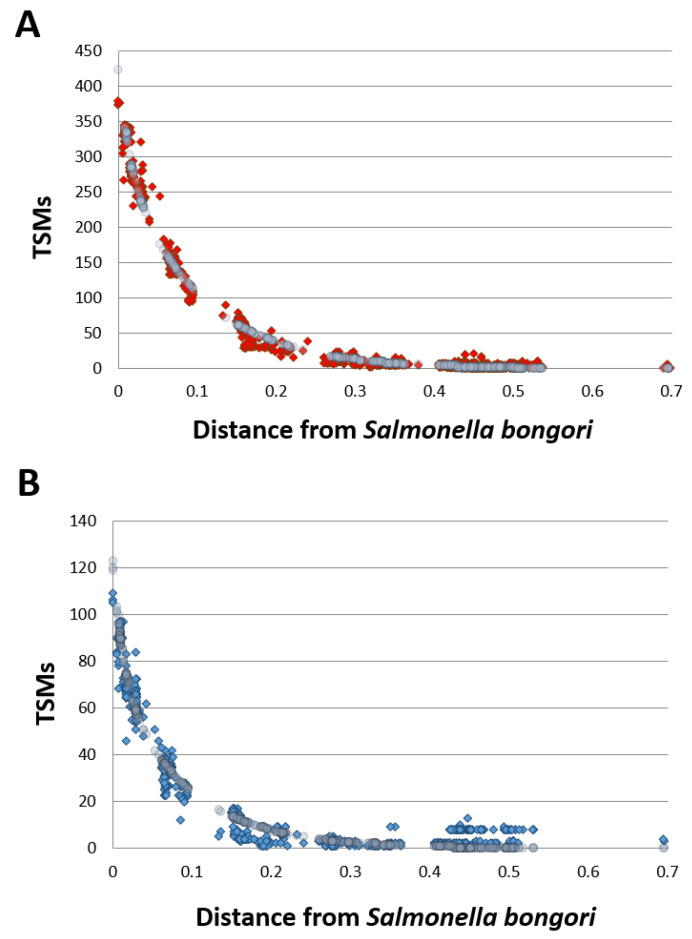
Phylopeptidomic signature of *S. bongori* harvested in exponential and stationary phases. **Panel A**: Exponential phase (replicate 1). Experimental TSMs (for each taxon present in the database) are indicated by red dots and the mathematical signature is represented by grey circles. **Panel B**: Stationary phase (replicate 1). Experimental TSMs (for each taxon present in the database) are indicated by blue dots and the mathematical signature is represented by grey circles. The calculated distance of each taxon present in the database (*x* axis) is indicated from *Salmonella bongori*. The phylogenetic distance is expressed as the ratio of differing residues between two taxa based on a multi-alignment of conserved COGs ubiquitous across superkingdoms and considering a total of 8310 amino acid positions as previously defined (Pible et al., 2020). A phylogenetic distance of 0.1 means that 10% of the 8310 amino acids are differing between the two taxa.

This approach based on both unique taxon-specific peptide sequences and TSMs circumvents possible false-positives when relying only on the former criterion alone. Figure 2 (Panel A) depicts the specific phylopeptidomic signature of this sample, which indicates the number of TSMs shared between *S. bongori* and all other organisms present in the NCBInr database and organized according to their respective phylogenetic distance from *S. bongori*. Logically, the shared TSMs decrease with phylogenetic distance since the average similarity between the peptide sequences of the organisms is inversely proportional to this distance. The signature fits the experimental points perfectly since only one species is present in the sample. Figure 2 (Panel B) shows the same phylopeptidomic signature for cells harvested in stationary phase. As shown in Table 1, *S. bongori* is the only validated species for all six samples. Although the proteomes differ between the exponential and stationary phases and, therefore the sets of peptides identified were dissimilar, enough unique taxon-specific peptide sequences and TSMs validated the species taxonomical rank, and similar phylopeptidomics signatures were obtained (Figure 2).

**Figure 3:**
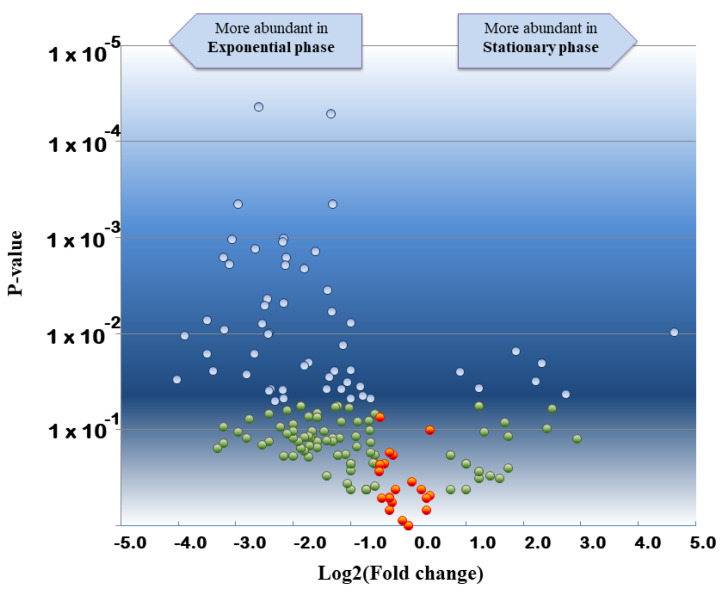
Volcano plot showing the comparative proteomics results of *S. bongori* stationary phase versus exponential phase. Tfold change was calculated taking into consideration the three biological replicates of both conditions. Proteins are indicated with blue circles if they satisfied both, the Tfold change (≥1.5 or ≤−1.5) and statistical criteria (*p*-value ≤ 0.05), orange circles for identifications that did not meet the fold criterion but have low *p*-values, green circles if they satisfied the fold criteria but, most likely, this happened by chance (*p*-value > 0.05), and red circles if they did not meet the fold and *p*-value criteria. The Benjamini–Hochberg test indicates that the FDR is less than 10% for this dataset.

Proteomic interpretation of the same dataset against the annotated *S. bongori* NTC 12419 genome revealed the detection of 1262 unique peptides and 6238 PSMs when the data from the 6 replicates were merged (Supplementary Materials, Table S1). These numbers are higher than those from the phylopeptidomic analysis because the database is much smaller. In total, 202 proteins could be validated with at least two different peptide sequences. As previously described for other bacteria [36], strong proteome differences are noted when comparing exponential and stationary phases. Indeed, only 55 proteins are consistently detected for the three exponential phase replicates and the three stationary phase replicates, whereas 92 and 18 are systematically only found in exponential phase and stationary phase, respectively (Supplementary Materials, Table S2). Moreover, the abundances of these proteins may differ considerably due to divergence in their metabolism. Figure 3 shows the volcano plot obtained when comparing the proteomes of the two growth stages. The most differentially abundant proteins in stationary phase are the DNA starvation/stationary phase protection protein [WP_000100806.1], the peptidoglycan-binding protein LysM [WP_015702978.1], and the outer membrane protein W [WP_000714794.1] with Tfold changes of 24.7, 6.7, and 5.0, respectively. The most differentially abundant proteins in the exponential phase are the 50S ribosomal protein L20 [WP_000124850.1], the DNA-directed RNA polymerase subunit beta [WP_000263100.1], the molecular chaperone DnaK [WP_000516126.1], and the lysine decarboxylase CadA [WP_001100654.1], with Tfold changes of −16.3, −14.8, −11.3, and −11.3, respectively. In conclusion, this analysis shows that phylopeptidomics is independent of bacterial growth stage and delivers the expected species if sufficient MS/MS were recorded.

### 3.2. Detection Limit of Proteotyping for Three Pure Microorganism Samples

The detection limit of tandem mass spectrometry proteotyping was assessed using three different species: *S. bongori*, *B. thuringiensis*, and *D. proteolyticus*. These bacteria belong to three distinct phyla, namely Proteobacteria, Firmicutes, and Deinococcus-Thermus, respectively. They exhibit distinct Gram staining due to their specific membranes and walls. For this evaluation, we recorded MS/MS spectra for a range of samples normalized to 1.0 mL volume and containing 4 × 10^3^ cfu, 8 × 10^3^ cfu, 1 × 10^4^ cfu, 2 × 10^4^ cfu, 4 × 10^4^ cfu, and 8 × 10^4^ cfu with independent biological triplicates. Table 2 reports the interpretation of proteotyping at genus and species taxonomical ranks with the corresponding number of TSMs and unique taxon-specific peptide sequences at the corresponding limit of detection. The datasets recorded for the three *S. bongori* replicates at 4 × 10^4^ cfu identify the correct species, whereas at 2 × 10^4^ cfu the mass spectrometry signal was not sufficient to certify the correct species. At this low concentration, the interpreted signal is rather weak with only 76, 76, and 49 TSMs for the three replicates. It only certifies the presence of Enterobacteriaceae at the taxonomical rank of the family with 22, 23, and 21 TSMs and 4, 5, and 6 unique taxon-specific peptide sequences.

The datasets recorded for the three replicates of *B. thuringiensis* at 8 × 10^4^ cfu identifies the correct species, but a weaker signal at lower bacterial amounts prevents identification of the correct species. The same is true for *D. proteolyticus*, although only two of the three replicates at 8 × 10^4^ cells gave the correct species. As expected, the limit of detection of the phylopeptidomics method depends on the protein biomass analyzed by tandem mass spectrometry and the recorded signal. Here, we analyzed three different bacteria with relatively different characteristics in terms of size and shape. First, *S. bongori*, formerly *Salmonella choleraesuis subsp. bongori subsp. nov.,* is a Gram negative, motile and non-spore forming rod that measures 0.7–1.5 µm in diameter by 2.0–5.0 µm in length [38,39], thus its average volume is 3.3 µm^3^. The vegetative cells of *B. thuringiensis* are rod-shaped, 2.0–5.0 µm long and about 1.0 µm wide [40], so its average volume is 2.7 µm^3^. Finally, *D. proteolyticus* cells are non-motile Gram-positive spheres, with an average diameter of 0.8 to 1.0 μm, occurring singly or as diplococci (pairs) and occasionally seen in tetrads [41]. Their average volume is therefore 0.38 µm^3^. The pair of *D. proteolyticus* cells that form a single cfu when sprayed on an agar plate has an equivalent volume of 0.76 µm3. If the protein biomass contribution of each type of bacterium can be roughly estimated using their volume, then the biomass contribution of 4 × 10^4^ cfu of *S. bongori* is 22% larger than 4 × 10^4^ cfu of *B. thuringiensis* (×1.2) and much larger than the biomass contribution of the same number of cfu of *D. proteolyticus* (×4.3). This biomass contribution explains why the detection limit for the three strains oscillates between 4 × 10^4^ and 8 × 10^4^ cfu and is revealed here to be dependent on the size and shape of the bacteria. Of course, this detection limit depends on the tandem mass spectrometer used for the analytical measurements, the parameters for the acquisition, the chromatography performances, the efficiency of the protein extraction protocol, which can vary depending on the cell type, as well as the amount of peptides that was injected. Here, only half of the protein extract was subjected to SDS-PAGE and proteolyzed.

### 3.3. Identification of Possible Biomarkers for Proteomic Detection of the Three Pure Microorganism Samples for Minute Loads

We interpreted the recorded datasets for the full range of cell quantities (6 conditions × 3 replicates × 3 strains) used in the experiment by a classical proteomics approach with a dedicated database. Table S3 lists the PSMs assigned in each condition for each microbial strain and Table S4 shows the detected proteins and their respective spectral counts per condition and strain. Logically, the number of proteins detected decreases with the cell biomass used for the analysis. For *S. bongori*, a total of 51, 36, 22, 7, 3, and 2 proteins were consistently detected for the three replicates for the samples containing 8 × 10^4^ cfu, 4 × 10^4^ cfu, 2 × 10^4^ cfu, 1 × 10^4^ cfu, 8 × 10^3^ cfu, and 4 × 10^3^ cfu, respectively. The three most detected proteins in these low biomass samples are different for the three bacteria. Elongation factors Tu [WP_000031748.1] and G [WP_000124707.1] and OmpA porin annotated as a membrane protein [WP_015702792.1] are consistently detected in *S. bongori* samples with at least two spectral counts in each replicate if the biomass was at least 2 × 10^4^ cfu. Only the first protein is detected from a biomass of 1 × 10^4^ cfu, but this protein is not consistently detected with at least two spectral counts for lower biomass amounts. For *B. thuringiensis*, the elongation factor Tu [WP_001029617.1], the 50S ribosomal protein L5 [WP_001080829.1], and the 30S ribosomal protein S2 [WP_000111485.1] were the most detected proteins. They accounted for at least two spectral counts systematically detected in each replicate with a biomass of at least 4 × 10^4^ cfu. The three most detected proteins for *D. proteolyticus* are a protein annotated as hypothetical but exhibiting sequence similarities with S-layer protein [WP_041221871.1], another S-layer domain-containing protein [WP_013614653.1], and an iron ABC transporter substrate-binding protein [WP_013615085.1]. In this case, a biomass of at least 8 × 10^4^ cfu is required to detect them with at least two spectral counts but only in two of three replicates. Based on these results, a targeted proteomics approach can be proposed to specifically quantify these most detected proteins by shotgun proteomics and most likely improve the detection limit of the three bacteria.

### 3.4. The Detection Limit of Phylopeptidomics Is Not Degraded in Multi-Organism Samples

**Figure 4:**
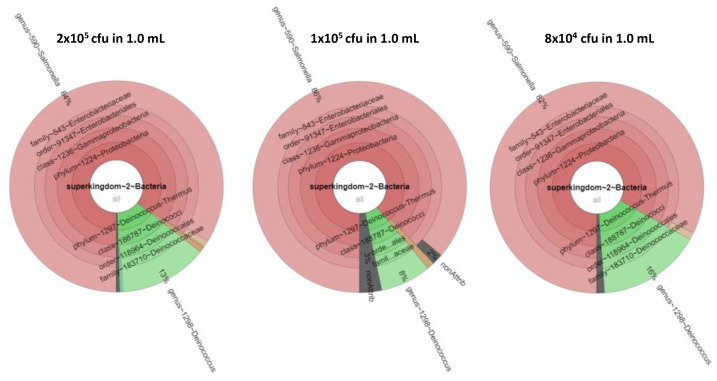
Krona presentation of the results of tandem mass spectrometry proteotyping. Identification results and biomass contributions of the three concentrations 2 × 10^5^ cfu, 1 × 10^5^ cfu, and 8 × 10^4^ cfu are shown from left to right. The biomass contributions of *S. bongori, D. proteolyticus*, and the unassigned signal are shown in red, green, and grey.

**Figure 5:**
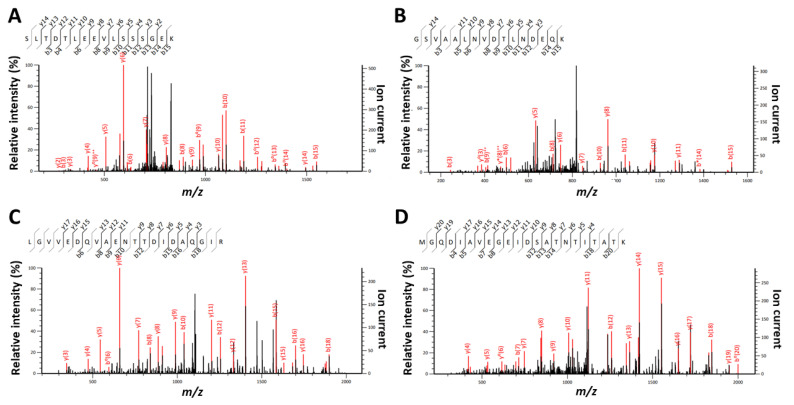
Representative MS/MS spectra and their MASCOT annotation for species-specific peptides. **Panel A**: MS/MS spectrum assigned to peptide SLTDTLEEVLSSSGEK (*m*/*z* 847.92169; z = 2; MASCOT score = 53.33) and specific of *S. bongori* species. **Panel B**: MS/MS spectrum attributed to peptide GSVAALNVDTLNDEQK (*m*/*z* 837.42096; z = 2; MASCOT score = 45.64) and specific of *S. bongori* species. **Panel C**: MS/MS spectrum #1669 (F404441 file) corresponding to peptide LGVVEDQVAENTTDIDAQGIR (*m*/*z* 1122.0614; z = 2; MASCOT score = 102.67) and specific of *D. proteolyticus* species. **Panel D**: MS/MS spectrum attributed to peptide MGQDIAVEGEIDSATNTITATK (*m*/*z* 1133.04687; z = 2; MASCOT score = 103.94) and specific of *D. proteolyticus* species. Ions indicated in red are those assigned and included in the MASCOT scoring.

Equimolar mixtures of *S. bongori* and *D. proteolyticus* were prepared at 2 × 10^4^ cfu, 4× 10^4^ cfu, 8 × 10^4^ cfu, 1 × 10^5^ cfu, and 2 × 10^5^ cfu in a 1.0 mL volume. In this case, both bacteria were successfully identified by phylopeptidomics when at least 8 × 10^4^ cfu were present in the sample, as shown in Table 3. At 4 × 10^4^ cfu, both bacteria were successfully identified in two of the three replicates, but at 2 × 10^4^ cfu, the bacteria were not detected. Noteworthy, the presence of additional peptides (those from *S. bongori* which is more abundant) improves the detection of *D. proteolyticus* peptides, probably because less loss of peptides from the tube walls occurred during extraction and chromatography of these low amounts of products. Indeed, single-cell proteomics often use an excess of peptides or proteins from unrelated species to promote a carrier effect and minimize adsorption losses on surfaces. Figure 4 shows the corresponding krona charts. As mentioned above, the biomass contributions in terms of protein amounts are different between the two types of bacteria. *S. bongori* is more abundant in these mixtures than *D. proteolyticus* due to differences in cell size and shape. Here, for the three replicates at 8 × 10^4^ cfu, *S. bongori* and *D. proteolyticus* are detected with 91/43/73 and 6/6/14 TSMs, respectively, while unique taxon peptides are 2/2/1 and 6/6/13, respectively. For the three replicates at 4 × 10^5^ cfu, *S. bongori* and *D. proteolyticus* are detected with 64/166/337 and 8/67/53 TSMs, respectively, while unique taxon peptides are 1/2/5 and 7/46/36, respectively. A great deal of heterogeneity is noted among the replicates, but basically, the ratio of *S. bongori* TSMs to *D. proteolyticus* TSMs is x7.9, x10.8, and x4.4 for the samples at 8 × 10^4^ cfu, 1 × 10^5^ cfu, and 2 × 10^5^ cfu, respectively. When sufficient bacteria are present, i.e., 2 × 10^5^ cfu, the observed ratio of TSMs is strikingly consistent with the theoretical biomass contributions inferred from the shape and size of both types of bacteria, i.e., ×4.3. Table S5 lists the PSMs assigned for these samples. Table S6 shows the proteins detected when using a dedicated database of limited size. Remarkably, the number of *D. proteolyticus* proteins validated with at least two specific peptides increased strongly in presence of *S. bongori*: 41 (Table S6) instead of 5 (Table S4). Indeed, the number of unique peptide sequences sharply enlarged: 278 (Table S5) instead of 78 (Table S3), confirming a decrease in peptide loss when the peptide load is higher. The most abundant proteins detected in pure samples are also found here among the most abundant entities. Figure 5 shows four representative MS/MS spectra assigned to peptide sequences that are species-specific for *S. bongori* and *D. proteolyticus*. A relatively good fragmentation is observed with most of their sequences covered by the *b* and *y* ions although the low peptide signal. Here, we choose to explore equimolar ratio of microorganisms in order to avoid any bias due to the different responsiveness of one proteome compared to others. In real life, mixed cultures may contain microorganisms at various ratios and as a result the limit of detection may be different depending on the asymmetry of the mixture.

## 4. Conclusions

Phylopeptidomics is a phylogeny-based approach which is peptide-centric. This is clearly different from metaproteomics interpretation tools which are protein-centric. We illustrated this approach combining unique taxon-specific peptide sequences and taxon-spectrum-matches with several bacteria: *S. bongori*, *B. thuringiensis*, and *D. proteolyticus*. Identification of bacteria using phylopeptidomics was found to be independent of their growth stage. The detection limit, expressed in cfu per mL, depends on the shape and size of the microorganisms as it is strongly correlated to the protein content. It varies between 4 × 10^4^ to 8 × 10^4^ cfu per mL, a detection limit remarkably relevant compared to targeted proteomics approaches limited to the abundance of specific peptides. Interestingly, the method is applicable to any type of microorganisms. In addition, shotgun proteomics may help define specific, but abundant, peptide biomarkers to improve the detection limit via targeted proteomics. Last, the detection limit of *D. proteolyticus* was improved in the presence of *S. bongori*. Based on the limit of detection described in this study, tandem mass spectrometry-based proteotyping has great potential.

## Figures and Tables

**Table 1:** Number of TSMs and specific peptides for *S. bongori* samples at 1 × 10^6^ cells.

Sample		Exponential Phase			Stationary Phase	
DAT file	F404181	F404182	F404183	F404187	F404188	F404189
#MS/MS spectra PSMs	4984	4987	4778	3554	2545	3101
	428	442	396	185	86	152
SUPERKINGDOM	Bacteria					
#TSMs #specific peptides	372	394	346	139	75	118
	197	211	179	79	38	68
PHYLUM	Proteobacteria					
#TSMs #specific peptides	368	390	341	136	69	113
	83	87	81	55	25	39
CLASS	Gammaproteobacteria					
#TSMs #specific peptides	365	389	340	135	67	113
	48	47	46	32	15	23
ORDER	Enterobacterales					
#TSMs #specific peptides	361	386	336	131	67	110
	40	38	42	31	14	23
FAMILY	Enterobacteriaceae					
#TSMs #specific peptides	361	386	336	131	67	110
	40	38	42	31	14	23
GENUS	*Salmonella*					
#TSMs #specific peptides	357	380	330	128	66	109
	14	14	14	8	4	10
SPECIES	*S. bongori*					
#TSMs #specific peptides	357	380	330	128	65	109
	4	4	5	3	1	3

**Table 2:** Number of TSMs and specific peptides for *S. bongori, B. thuringiensis, and D. proteolyticus* samples at their respective limit of detection (4 × 10^4^, 8 × 10^4^, 8 × 10^4^ cfu, respectively).

Sample *S. bongori* 4 × 10^4^	Replicate 1	Replicate 2	Replicate 3
DAT file	F404017	F404018	F404019
#MS/MS spectra	3953	3563	2752
#PSMs	115	108	73
GENUS	*Salmonella*	*Salmonella*	*Salmonella*
#TSMs	60	62	27
#specific peptides	3	3	1
SPECIES	*S. bongori*	*S. bongori*	*S. bongori*
#TSMs	60	62	27
#specific peptides	0	0	0
Sample *B. thuringiensis* 8 × 10^4^	Replicate 1	Replicate 2	Replicate 3
DAT file	F404340	F404341	F404342
#MS/MS spectra	3122	2942	3468
#PSMs	73	62	115
GENUS	*Bacillus*	*Bacillus*	*Bacillus*
#TSMs	29	39	93
#specific peptides	5	5	15
SPECIES	*B. thuringiensis*	*B. thuringiensis*	*B. thuringiensis*
#TSMs	29	38	90
#specific peptides	1	1	3
Sample *D. proteolyticus* 8 × 10^4^	Replicate 1	Replicate 2	Replicate 3
DAT file	F404314	F404315	F4043116
#MS/MS spectra	1886	1483	1934
#PSMs	16	14	38
GENUS	*Deinococcus*	*Deinococcus*	*Deinococcus*
#TSMs	/	7	16
#specific peptides	/	7	15
SPECIES	*D. proteolyticus*	*D. proteolyticus*	*D. proteolyticus*
#TSMs	/	7	16
#specific peptides	/	7	15

**Table 3:** Number of TSMs and specific peptides for *S. bongori* and *D. proteolyticus* mixed samples at 8 × 10^4^ cfu.

Sample	Replicate 1		Replicate 2		Replicate 3	
DAT file	F404367		F404368		F404369	
#MS/MS spectra #PSMs	2779		3098		2409	
	124		113		103	
GENUS	*Salmonella*	*Deinococcus*	*Salmonella*	*Deinococcus*	*Salmonella*	*Deinococcus*
#TSMs #specific peptides	92	6	43	6	73	14
	7	6	3	6	2	14
SPECIES	*S. bongori*	*D. proteolyticus*	*S. bongori*	*D. proteolyticus*	*S. bongori*	*D. proteolyticus*
#TSMs #specific peptides	91	6	43	6	73	14
	2	6	2	6	1	13

## Data Availability

Data are available in supplementary Tables S1–S6 provided together with the main publication.

## Supplementary Materials

The following supporting information can be downloaded at: https://www.mdpi.com/article/10.3390/microorganisms11051170/s1, Table S1: List of PSMs for *S. bongori* at 1 × 10^6^ in exponential and stationary phases. Table S2: List of identified proteins for *S. bongori* at 1 × 10^6^ in exponential and stationary phases. Table S3: List of PSMs for *S. bongori, B. thuringiensis*, and *D. proteolyticus* at 4 × 10^3^ cfu, 8 × 10^3^ cfu, 1 × 10^4^ cfu, 2 × 10^4^ cfu, 4 × 10^4^ cfu, and 8 × 10^4^ cfu. Table S4: List of identified proteins for *S. bongori*, *B. thuringiensis,* and *D. proteolyticus* at 4 × 10^3^ cfu, 8 × 10^3^ cfu, 1 × 10^4^ cfu, 2 × 10^4^ cfu, 4 × 10^4^ cfu, and 8 × 10^4^ cfu. Table S5: List of PSMs for *S. bongori* and *D. proteolyticus* mixtures at 2 × 10^4^ cfu, 4 × 10^4^ cfu, 8 × 10^4^ cfu, 1 × 10^5^ cfu, and 2 × 10^5^ cfu. Table S6: List of identified proteins for *S. bongori* and *D. proteolyticus* mixtures at 2 × 10^4^ cfu, 4 × 10^4^ cfu, 8 × 10^4^ cfu, 1 × 10^5^ cfu, and 2 × 10^5^ cfu.
